# Renal auto-transplantation promotes cortical microvascular network remodeling in a preclinical porcine model

**DOI:** 10.1371/journal.pone.0181067

**Published:** 2017-07-13

**Authors:** Souleymane Maïga, Geraldine Allain, Thierry Hauet, Jerome Roumy, Edouard Baulier, Michel Scepi, Manuel Dierick, Luc Van Hoorebeke, Patrick Hannaert, Franck Guy, Frederic Favreau

**Affiliations:** 1 INSERM, U1082, Poitiers, France; 2 Universités de Poitiers, Faculté de Médecine et de Pharmacie, Poitiers, France; 3 Service d'Imagerie Diagnostique et Interventionnelle de l'Adulte, Centre Hospitalier Universitaire de Bordeaux, Groupe Hospitalier Pellegrin, Bordeaux, France; 4 CHU de Poitiers, Service de Chirurgie Cardio-Thoracique, Poitiers, France; 5 CHU de Poitiers, Laboratoire de Biochimie, Poitiers, France; 6 INRA, UE1372 GenESI, Plateforme IBISA, Surgères, France; 7 Fédération Hospitalo Universitaire de Transplantation, SUrvival oPtimization in ORgan Transplantation (SUPORT), Limoges, Poitiers and Tours, CHU La Milétrie, Poitiers, France; 8 UGCT-Department of Physics and Astronomy, Ghent University, Faculty of Sciences, Proeftuinstraat 86, Ghent, Belgium; 9 CNRS INEE UMR 7262, IPHEP Institut de Paléoprimatologie et Paléontologie Humaine, Évolution et Paléoenvironnements, Poitiers, France; Universités de Poitiers, Faculté des Sciences, Poitiers, France; Universita degli Studi di Torino, ITALY

## Abstract

The vascular network is a major target of ischemia-reperfusion, but has been poorly investigated in renal transplantation. The aim of this study was to characterize the remodeling of the renal vascular network that follows ischemia-reperfusion along with the most highly affected cortex section in a preclinical renal transplantation model. There were two experimental groups. The first was a grafted kidney group consisting of large white pigs for which the left kidney was harvested, cold flushed, preserved for 24 h in the University of Wisconsin’s preservation solution, and then auto-transplanted (n = 5); the right kidney was removed to mimic the situation of human kidney transplantation. The second group (uni-nephrectomized kidney group) consisted of animals that underwent only right nephrectomy, but not left renal transplantation (n = 5). Three months after autotransplantation, the kidneys were studied by X-ray microcomputed tomography. Vessel morphology and density and tortuosity of the network were analyzed using a 3D image analysis method. Cortical blood flow was determined by laser doppler analysis and renal function and tissue injury assessed by plasma creatinine levels and histological analysis. Renal ischemia-reperfusion led to decreased vascular segment volume associated with fewer vessels of less than 30 μm, particularly in the inner cortex:0.79 ± 0.54% in grafted kidneys *vs*. 7.06 ± 1.44% in uni-nephrectomized kidneys, p < 0.05. Vessels showed higher connectivity throughout the cortex (the arborescence factor of the whole cortex was less in grafted than uni-nephrectomized kidneys 0.90 ± 0.04 *vs*. 1.07 ± 0.05, p < 0.05, with an increase in the number of bifurcations). Furthermore, cortical blood flow decreased early in kidney grafts and remained low three months after auto-transplantation. The decrease in microvasculature correlated with a deterioration of renal function, proteinuria, and tubular dysfunction, and was associated with the development of fibrous tissue. This work provides new evidence concerning the impact of ischemia-reperfusion injuries on the spectrum of renal vascular diseases and could potentially guide future therapy to preserve microvessels in transplantation ischemia-reperfusion injury.

## Introduction

The vascular microcirculation network is a major target in numerous cardiovascular disorders, in particular ischemia-reperfusion (IR) following renal transplantation [1]. In this condition, harvesting of the kidney induces ischemia and grafting induces reperfusion. IR syndrome can result in delayed and/or chronic graft dysfunction to nonfunctioning of the primary graft, depending on the degree of kidney damage, requiring the patient to return to dialysis [2].

Endothelial cells and the microvascular network [3] are critical targets of IR. Renal microcirculation plays a crucial role in the early phase of the transplantation process, due to its high sensitivity to hypoxic conditions [4, 5]. During the development of chronic graft damage, renal microcirculation is also affected by complex interactions between proliferation, regeneration, and capillary loss, leading to fibrosis, considered to be the major process responsible for late renal graft loss.

Renal microvasculature has classically been studied using methods such as pharmacological modulation, vascular filling techniques, light microscopy, micro-angiography, and scanning electron microscopy [6, 7]. High-resolution microcomputed tomography (HR-μCT) is a powerful tool for studying the architecture of 3D vasculature in numerous experimental pathophysiological situations [8–12]. Yet, this technique has never been exploited to study the effects of cold storage in the transplantation process. The importance of using 3D measuring techniques was highlighted by Minnich *et al*., who compared the accuracy of 2D and 3D measurements of microvasculature lengths. They showed 3D morphometry to be the method of choice for length measurement [13]. Furthermore, 2D techniques suffer from limited sample thickness and poor depth of analysis [14]. HR-μCT has been compared to light microscopy for characterization of the hierarchical structure of vascular networks in previous studies. It has proven to be an accurate measurement technique for interbranch segment lengths and diameters [15]. Here, we studied the renal vascular network in a porcine renal transplantation model using 3D HR-μCT imaging and histology. We focused on the consequences of renal vascular network remodeling on renal blood flow and graft function.

## Materials and methods

### Animal care and use

Animal experimental procedures were performed in accordance with the guidelines of the French Ministries of Agriculture and Research and approved by the institutional committee for the use and care of laboratory animals (CEEA Poitou-Charentes, project reference number: CE2012-4). After an adaptation period of three days, 10 large white three-month-old male pigs, weighing 30 to 35 kg, from the pig breeding program of the National Institute of Agronomic Research (Le Magneraud, Surgères, France), were prepared as previously described [16, 17]. Briefly, they were maintained in a climate-controlled room at a temperature of 22 ± 2°C and a humidity of 60 ± 5%, on a light/dark cycle, and provided a standard commercial diet (Arrive Society, Saint Fulgent, France) and water *ad libitum*. During the seven days after surgery, they were housed in individual boxes to allow rapid intervention to maintain analgesia. During the three-month follow up, they were housed in individual boxes near the other pigs and placed in metabolic cages 1, 3, 5 7, and 14 days, and 1 and 3 months after surgery to perform blood and 24-h-urine collection.

### Anesthetic and analgesic protocols and euthanasia

The animals were fasted for 24 h before surgery. The induction of anesthesia was performed with a Hunter mask using a mixture of nitrous oxide and oxygen (50/50) associated with 8% sevoflurane and then 2.5% isoflurane. A perfusion line was set in a marginal vein of the ear. Orotracheal intubation was performed under laryngoscopic control. Controlled ventilation was performed with a respiratory rate of 16/min while maintaining expired CO_2_ between 35 and 45 mmHg. Curarisation of the animal was performed by the initial injection of 8 mg pancuronium bromide followed by reinjection adapted to the behavior of the animal. Intraoperative analgesia was achieved by intravenous injections of 20 mg nalbuphine and 20 mg nefopam and antibioprophylaxis by intravenous administration of amoxicillin-clavulanic acid (1 g) at the beginning of the procedure. During follow up, analgesia was maintained by intravenous injection of 20 mg nalbuphine per day for the first three days, antibioprophylaxis by the intravenous administration of 1g amoxicillin-clavulanic acid, per day, for the first seven days, and prophylaxis against stomach ulcer by intravenous administration of 40 mg pantoprazole per day for the first five days. The animals were euthanized three months after surgery. Induction of anesthesia was performed with a Hunter mask as already described, the left kidney removed through a subcostal approach, and euthanasia achieved by intravenous injection of 2 g potassium chloride.

### Surgical procedure

For harvesting of the left kidney, the animal was placed in a supine position with access to the cervical and abdominal regions. A central venous catheter was positioned in the internal jugular vein after left cervicotomy. It was externalized behind the left ear and kept in place for 14 days post-transplantation for the administration of drugs and blood sampling. After a median laparotomy, the left kidney was removed extra-peritoneally. It was cold flushed and preserved at 4°C for 24 h using the University of Wisconsin’s preservation solution. The laparotomy was closed and the animal extubated and awakened. During the left kidney transplantation procedure, the laparotomy was reopened and the right kidney removed transperitoneally. The abdominal aorta and sub-renal inferior vena cava were dissected on the left side to prepare the graft site. Arterial and then venous termino-lateral anastomosis were performed above the iliac bifurcation. Declamping was performed 30 min after the beginning of the anastomoses and uretero-vesical anastomosis was performed and a double J-catheter installed. The graft was positioned retro-peritoneal and the posterior parietal peritoneum closed. The laparotomy was closed and the animal extubated and awakened.

### Experimental groups

Experimental groups consisted of i) a grafted kidney group: animals for which the left kidney was harvested, cold flushed, preserved at 4°C for 24 h, and then auto-transplanted; the contralateral kidney was removed to mimic the situation of a human kidney transplantation (n = 5); ii) a uni-nephrectomized kidney group: animals that underwent right nephrectomy, but not left renal transplantation (n = 5).

### Monitoring of renal function

Plasma and urinary creatinine, sodium, and magnesium levels, as well as proteinuria, were measured using an automated chemistry analyzer (Modular, Roche Diagnostics, Meylan, France). The osmolarity ratio (blood/urine) was assessed by the freezing point depression method with an osmometer (Hermann Roebling, Messtechnik, Berlin, Germany).

### Tissue preparation and image acquisition

Immediately after removal, the left kidney was perfused, using gravity, through the main renal artery with a saline solution containing 5,000 IU/L heparin for 10 min. The saline solution was then replaced by a freshly mixed intravascular radio-opaque silicone polymer (Microfil MV122; Flow Tech, Carver, MA), which was injected at constant pressure until the polymer drained freely from the segmental vein (1.65 mL/min). The kidney was then immersed in formalin solution at 4°C. A cylindrical biopsy (diameter: 9 mm; depth: 15 mm) of the polymer-filled kidney was carried out from the tip of the renal lower lobe using a punch and encased in paraffin. The lower pole was chosen because it is known to be most commonly perfused by a single segmental artery [18]. The HR-μCT device was composed of a Feinfocus transmission type X-ray tube, with a maximum resolution of 900 nm, an air-bearing rotation stage, and a Varian Paxscan flat-panel detector (Varian, Palo Alto, California, USA). The following acquisition parameters were used: tube voltage of 120kV, 3 mm aluminum beam filtration, and 14-watt beam power. A standard cone-beam acquisition was recorded with 1,000 exposures of 2 s each, covering 360 degrees, with a voxel size of 21.9 μm. For scans and analyses, the operators were uninformed of the experimental conditions.

### Vascular network analysis

**Fig 1:**
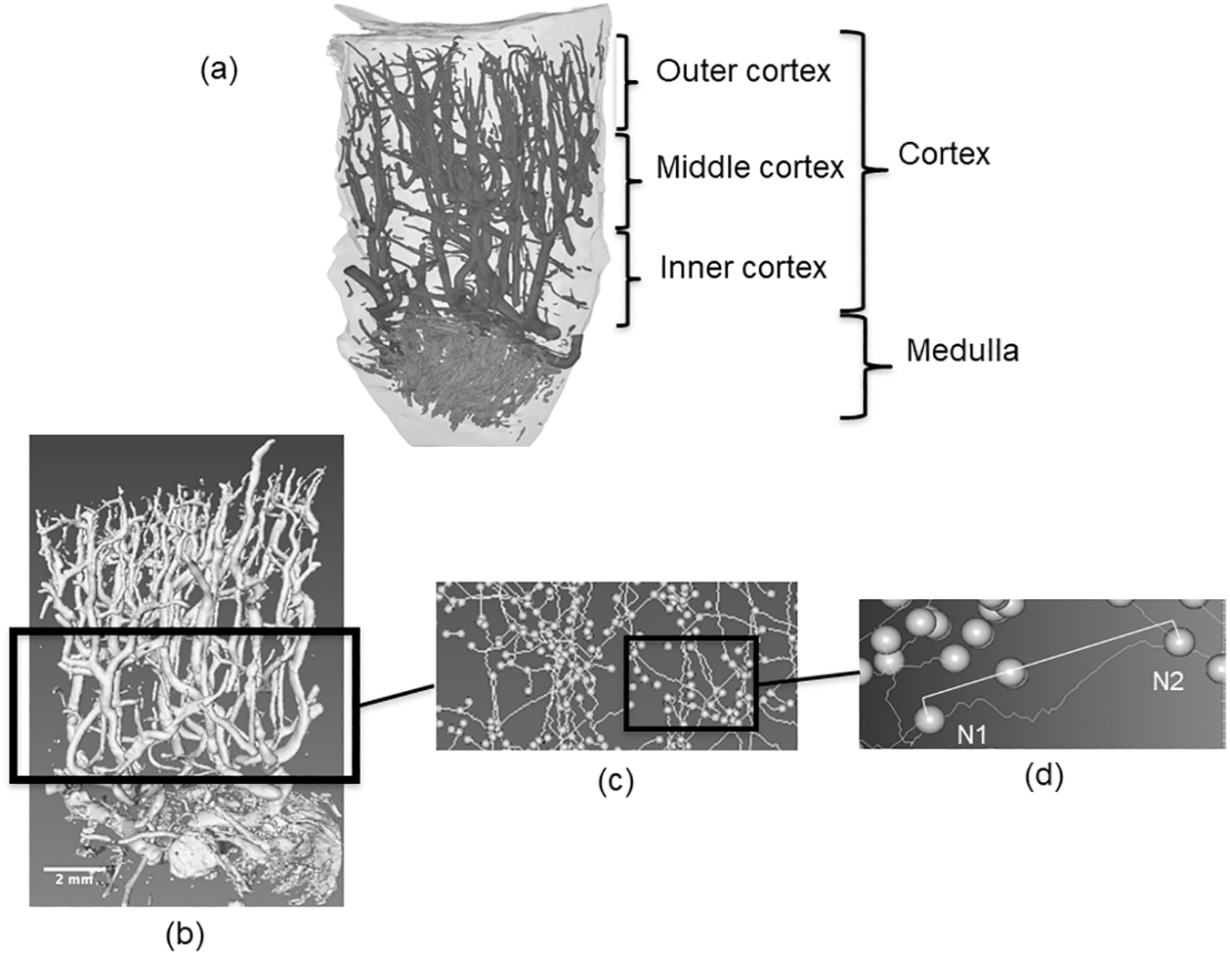
Vascular network analysis. (A) Subdivision of the renal cortex structure in the outer, middle, and inner cortex. (B) Volume rendering of the vascular network from the renal cortex. (C) Centerline skeletonization showing the nodes (spheres) and vascular segments (lines). (D) Example of a vascular segment between two nodes (N1 and N2). Tortuosity is the ratio between the curvilinear distance between N1- N2 and the respective chord linear distance.

The HR-μCT images were filtered at a threshold, using Image J software, to generate binary images of the vascular network and total tissue volume [19] and the segmented vascular network volume determined. The segmented vascular network volume dataset was used as the input for determining the centerline or “skeleton” of the vascular network using Avizo 7 Standard® software (Visualization Sciences Group, Zuse Institute Berlin, Germany). This was the first step in defining the vascular network topology. The centerline extraction method utilizes the TEASAR algorithm [20]. The module extracted the centerlines of a labelfield object. It searched for a graph with tree topology and did not generate loops. The Euclidean distance to the nearest boundary (boundary distance map) was stored at every point in the Spatial Graph object as a thickness attribute. This value may be used as an estimate of the local thickness. Considering locations along the centerline within the segmented image the hierarchical nature of the vascular network, segment curvilinear lengths, and segment diameters could subsequently be determined. Specific points of the centerline describing vessel dichotomy or trichotomy are called nodes and a vascular segment was defined by a portion of a vessel situated between two nodes (Fig 1A–1D). A node is either the start or termination of a segment or an intersection between three or more segments. Chord length was defined as the shortest linear distance between two nodes. Data processing was performed using R software (R Development Core Team, Foundation for Statistical Computing, Vienna, Austria).

#### Studied parameters

Cortical thickness was calculated as the number of sections perpendicular to the z-axis between the medulla and the outer surface, multiplied by slice thickness (21.9 μm). Based on the number of cortical slices, the cortex was then divided into three parts of equivalent thickness (Fig 1A) named the outer, middle, and inner cortex (1 to 3, respectively). The inner cortex was considered to be the cortico-medullary junction of the kidney. The vascular volume fraction was calculated as the fraction of total tissue volume of the sample corresponding to the vascular network volume. The average volume, length, and diameter of the vascular segment were each computed from the vascular network divided by the number of segments. The number of vessels was computed as a function of their diameters, which ranged from less than 30 to more than 120 μm. Tortuosity was defined as the ratio between the curvilinear length of a segment between two nodes and the associated chord linear length of the segment (Fig 1B–1D). The arborescence of the vascular network was defined as the ratio of the total length of the connected vascular network to the number of vascular segments. The number of bifurcations was counted to account for the complexity of the vascular network.

### Laser doppler flowmetry

Blood cell flow and velocity and the concentrations of moving blood cells were determined *in situ* in anesthetized animals, 60 minutes (reperfusion) and three months after transplantation, using a laser doppler (Periflux System 5000 Perimed, KB, Jarfalla, Sweden). Cortical perfusion in all animals was measured by systematically positioning the probes on the parenchyma in the middle portion of the kidney. The system was calibrated to a special motility standard provided by Perimed to enable comparison of the results. Values were recorded for 10 min after the placement of the probe.

### Immunohistochemistry and western blot analysis

*In vitro* studies were performed to assess the mechanisms responsible for the diminution or maintenance of the renal microvasculature, as well as the profibrotic and antiangiogenic factors following transplantation. Staining of the cortex samples was performed as previously described [16]. Briefly, paraffin sections were used to evaluate tubulo-interstitial fibrosis following red Sirius staining or alpha-smooth muscle actin expression with an alpha-SMA antibody (Ref: M0851, 1/100, Dako, Sweden) and an appropriate HRP-coupled secondary antibody (Ref: K4000, Dako, Sweden) revealed by diamino benzidine staining. The microvascular media-to-lumen ratio was measured in αSMA-positive microvessels under 500 μm in diameter. Frozen sections were used to assess CD31 positive cell staining by immunofluorescence (Ref: MCA1746F, CD31 antibody, 1/50, AbD Serotec, Oxford, UK) in the assessment of the decrease in the number of microvessels. Paraffin sections were used for quantifying aminopeptidase P-positive blood microvessels using an aminopeptidase P antibody (Ref SC-65390, 1/100, Santa Cruz Biotechnology, Santa Cruz, California, USA) and an appropriate HRP-coupled secondary antibody revealed by diamino benzidine staining. Protein levels were determined by western blotting, according to standard protocols [21], using samples from frozen cortical kidney biopsies and antibodies against transforming growth factor β (TGF β, Ref: SC-52981, 1/600), pro-matrix metalloproteinase 2 (Pro-MMP-2, Ref: SC-10736, 1/200), thrombospondin-1 (TSP-1, Ref: SC-12312, 1/200, all from Santa Cruz Biotechnology, Santa Cruz, California, USA), hypoxia inducible factor (HIF1a, 1/500 dilution, BD Biosciences, San Jose, California, USA), heme oxygenase-1 (HO-1, Ref: bs-2075R, 1/500, Bioss, Woburn, Massachusetts, USA), and β-actin (1/3000; Sigma, St Louis, Missouri, USA) as a loading control.

### NO production

Nitric oxide (NO) production was indirectly evaluated by quantification of NO metabolites (Nitrite+Nitrate) in homogenate from renal cortex samples (1/10 in phosphate buffered saline) using a colorimetric assay, according to the manufacturer’s instructions (Cayman Chemical, Ann Arbor, Michigan, USA).

### Statistical analysis

A sample size of five pigs per group was calculated based on a 2-sided α of 0.05, a power (1-β error probability) of 0.80, and an effect size of 2.1, based on our main criterion which was a decrease in the number of vessels with a diameter < 30 μm (G*Power software 3.1) [22]. Statistical analysis was performed using NCSS 2007 for Windows (Hintze, J. 2007, NCSS, LLC. Kaysville, UTAH). Results are given as the mean ± SEM. For statistical analysis, a non-parametric Mann Whitney test was used to compare both experimental groups. Correlation analysis was performed using NCSS software and is displayed as r^2^. Significance was established with the Spearman rank test. Results were considered to be statistically significant for p < 0.05.

## Results

### Renal function

**Fig 2:**
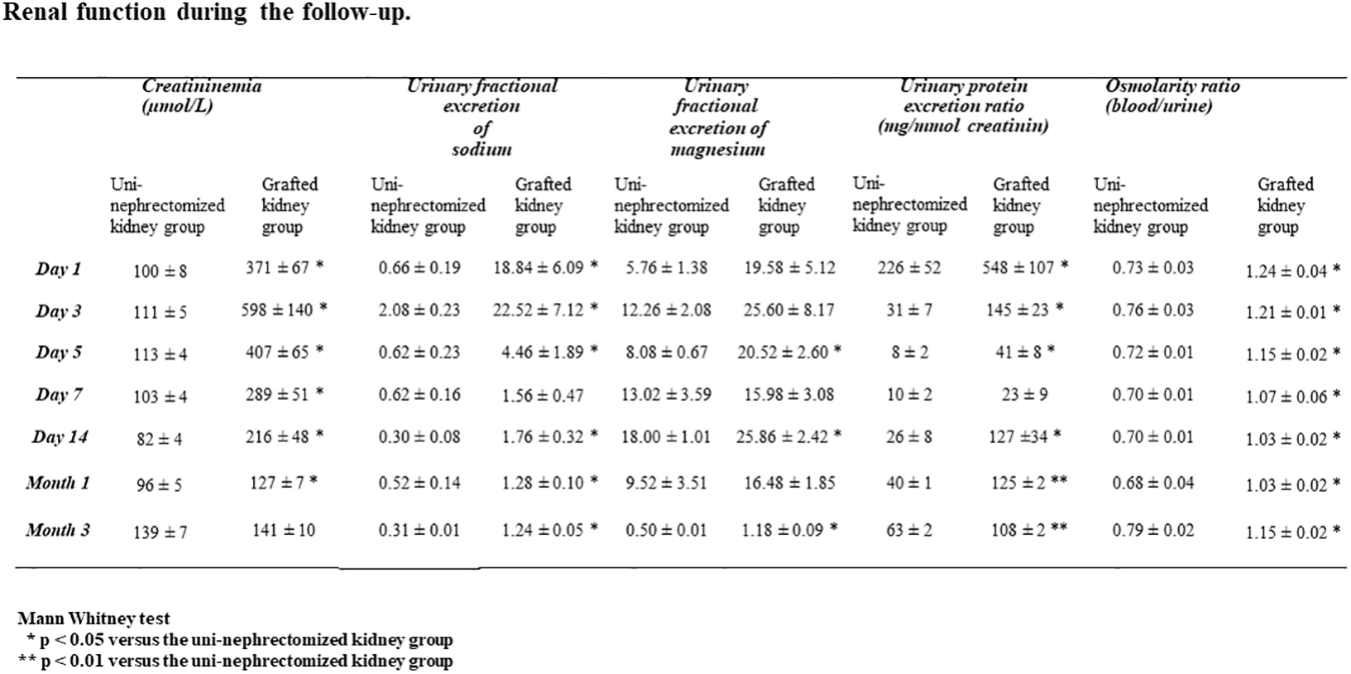
Renal function during the follow-up. Summary of the renal function evaluation parameters during the follow-up including plasma creatinine levels (μmol/L), urinary fractional excretion of sodium and magnesium, urinary protein excretion (mg/mmol creatinine), and osmolarity ratio (blood/urine). Values are expressed as the mean ± SEM (* p < 0.05 or ** p < 0.01 *vs*. the uni-nephrectomized kidney group).

All pigs survived the procedure to the termination point. Renal function, evaluated by plasma creatinine levels, did not significantly differ between either experimental group (139 ± 7 μmol/L for the uni-nephrectomized kidney group *vs*. 141 ± 10 μmol/L for the grafted kidney group three months after transplantation; p = NS; Fig 2 and S1 Appendix). We observed no visual signs of poor graft condition at the time of euthanasia. However, urinary fractional excretion of sodium and magnesium and osmolarity (blood/urine) ratios were all affected by transplantation after three months: 1.24 ± 0.05 *vs*. 0.31 ± 0.01 for sodium, 1.18 ± 0.09 *vs*. 0.50 ± 0.01 for magnesium, and 1.15 ± 0.02 *vs*. 0.79 ± 0.02 for osmolarity in the grafted kidney and uni-nephrectomized kidney groups, respectively (Fig 2, p < 0.05). The ratio of these levels correlated with the density of vessels with a diameter < 30 μm: r^2^ = 0.78, r^2^ = 0.68, and r^2^ = 0.72, respectively (p < 0.05).

### Ischemia reduces microcirculation in kidney grafts within three months

**Fig 3:**
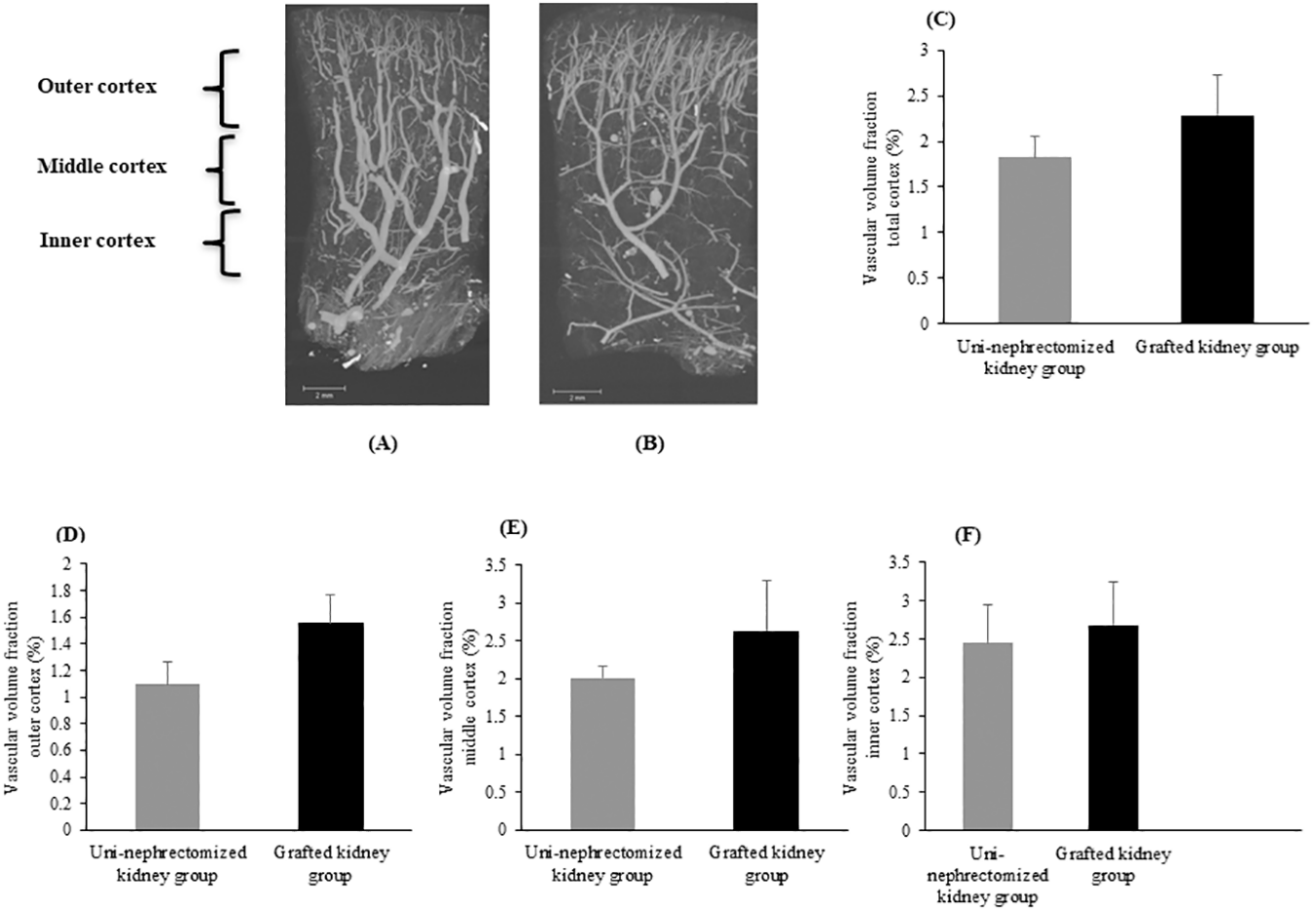
Histogram of vascular volume fractions. Coronal maximum intensity projection of the vascular network from renal cortex samples: A) Uni-nephrectomized kidney and (B) grafted kidney. Histogram of vascular volume fraction as the ratio between vascular network volume and total sample volume expressed as the percentage in samples of (C) total renal cortex, (D) the outer cortex, (E) middle cortex, and (F) inner cortex from the uni-nephrectomized and grafted kidney groups. Values are expressed as the mean ± SEM.

**Fig 4:**
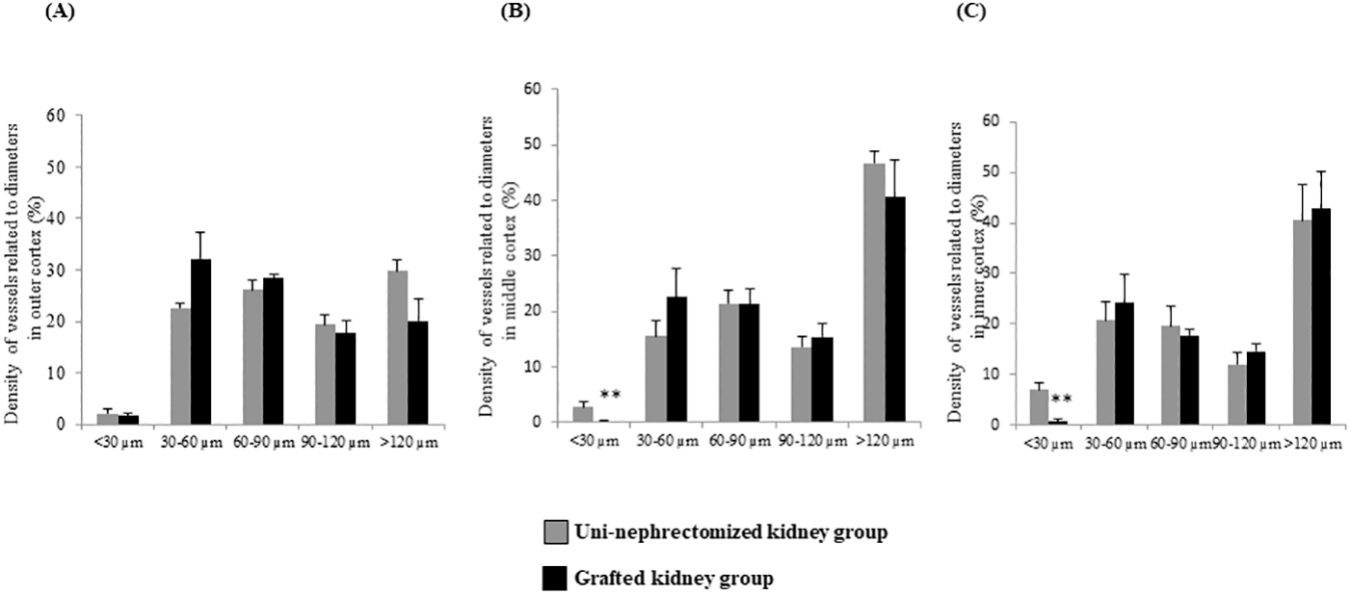
Ischemia reduces microcirculation in kidney grafts within three months. (A) Histogram of vessel density computed by vascular segment diameter expressed as a percentage of total vessels in the outer cortex, (B) middle cortex, (C) and inner cortex. Values (mean ± SEM) that differ significantly from those of the uni-nephrectomized kidney group are represented by **p < 0.01.

Three months after transplantation, cortical thickness was similar for the grafted kidney and uni-nephrectomized kidney groups (12.29 ± 0.45 mm *vs*. 11.41 ± 0.39 mm, p = NS, Table 1 and S1 Appendix), as well as the total vascular volume fraction (2.29 ± 0.44% *vs*.1.83 ± 0.23% in the total cortex, p = NS, and the three cortical regions; Fig 1A and Fig 3A–3F). However, the mean vascular segment volume in the whole cortex was significantly lower in grafted than uni-nephrectomized kidneys (13 ± 2.10^6^ μm^3^ *vs*. 30 ± 5.10^6^ μm^3^, p < 0.05, Table 1). We also observed this significant difference in the outer and middle cortex (p < 0.05 for both, Table 1). The length of the vascular segments was significantly less in the total cortex of grafted than uni-nephrectomized kidneys (902 ± 36 μm *vs*. 1075 ± 49 μm, p < 0.05, Table 1 and S1 Appendix). This was also true for the outer cortex (878 ± 33 μm *vs*.1,006 ± 41 μm, p < 0.05, Table 1). The density of vessels with a diameter > 30 μm did not differ between the two groups, but vessel density for segments < 30 μm in the whole cortical zone was significantly less in the grafted than uni-nephrectomized kidneys (0.55 ± 0.15% *vs*. 3.96 ± 0.67%, respectively, p < 0.05). This significant difference was also present in the middle (0.16 ± 0.10% *vs*. 2.72 ± 0.97%, p < 0.05, Fig 4A and 4B) and inner cortex (0.79 ± 0.54% *vs*. 7.06 ± 1.44%, p < 0.05, Fig 4C).

**Table 1:** Analysis of vascular segments and vascular network complexity in kidneys three months after autotransplantation.

Parameters	Uni-nephrectomized kidneys	Grafted Kidneys
**Cortical thickness (.10**^**3**^ **μm)**	**12.29 ± 0.45**	**11.41 ± 0.39**
**Vascular segment diameter (μm)**	**126 ± 8**	**108 ± 9**
outer cortex	101 ± 3	86 ± 6
middle cortex	146 ± 8	118 ± 11
inner cortex	142 ± 22	129 ± 14
**Length of vascular segment (μm)**	**1075 ± 49**	902 ± 36*
outer cortex	1006 ± 41	878 ± 33*
middle cortex	982 ± 81	857 ± 31
inner cortex	914 ± 50	912 ± 57
**Vascular segment volume (.10**^**6**^ **μm**^**3**^**)**	**30 ± 5**	13 ± 2*
outer cortex	12 ± 1	7 ± 1*
middle cortex	30 ± 5	13 ± 2*
inner cortex	35 ± 9	20 ± 3
**Arborescence factor**	**1.07 ± 0.05**	0.90 ± 0.04*
outer cortex	1.01±0.04	0.88±0.03*
middle cortex	0.98±0.08	0.86±0.03
inner cortex	0.91±0.05	0.91±0.06
**Tortuosity**	**1.21 ± 0.01**	**1.20 ± 0.01**
outer cortex	1.22 ± 0.01	1.22 ± 0.01
middle cortex	1.19 ± 0.01	1.19 ± 0.01
inner cortex	1.21 ± 0.01	1.19 ± 0.01
**Number of bifurcations**	**139 ± 26**	296 ± 43*
outer cortex	45 ± 13	93 ± 10*
middle cortex	52 ± 9	112 ± 22
inner cortex	42 ± 8	78 ± 14*

Mann Whitney test *p < 0.05, versus uni-nephrectomized kidney group.

### Ischemia promotes higher connectivity in the vascular network

Tortuosity did not significantly differ between kidney groups. The arborescence factor was lower in the kidney graft than uni-nephrectomized kidney group for the whole cortex (0.90 ± 0.04 *vs*. 1.07 ± 0.05, p < 0.05, Table 1 and S1 Appendix) and the outer cortex (0.88 ± 0.03 *vs*. 1.01 ± 0.04, p < 0.05, Table 1). These results were supported by a significantly larger number of bifurcations in the total, outer, and inner cortex of the grafted kidneys than those of the uni-nephrectomized kidneys (p < 0.05, Table 1).

### Cortical blood flow is impaired by IR

The laser doppler study revealed a significantly lower flow of blood cells in the renal cortex of grafted kidneys, 60 minutes and three months after reperfusion (both with p < 0.05 *vs*. uni-nephrectomized kidneys) (Table 2). Velocity levels and moving blood cell concentration showed a similar pattern (Table 2 and S1 Appendix).

**Table 2:** Analysis of cortical blood flow *in vivo* by laser doppler flowmetry in kidneys 60 minutes and three months after reperfusion (n = 4 in each experimental group).

Parameters (% of control values)	Uni-nephrectomized kidneys	Grafted kidneys
***1 hour***		
Blood cell flow	93.9 ± 1.5	12.2 ± 0.8 *
Velocity	98.3 ± 1.4	41.5 ± 0.5 *
Moving blood cells concentrations	92.0 ± 3.5	25.9 ± 0.2*
***3 months***		
Blood cell flow	91.3 ± 0.2	63.5 ± 0.4 *
Velocity	98.9 ± 0.7	70.7 ± 0.6 *
Moving blood cells concentrations	92.2 ± 1.1	52.2 ± 1.1*

Mann Whitney test * p < 0.05

### Renal transplantation induces vascular remodeling and interstitial fibrosis

**Fig 5:**
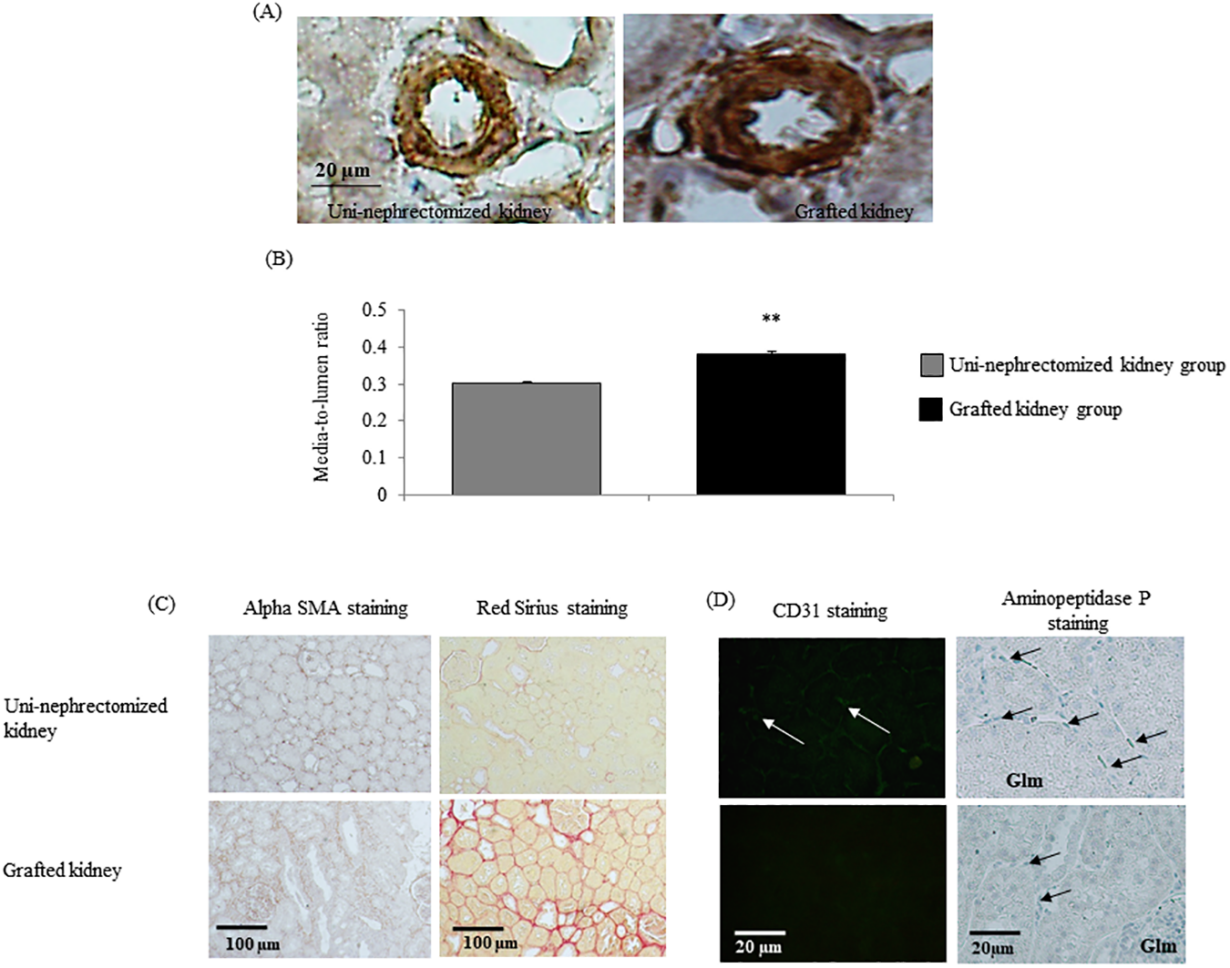
Renal transplantation induces vascular remodeling and interstitial fibrosis. (A-B) Media-to-lumen ratio of renal cortex samples of uni-nephrectomized and grafted kidneys. (C) Evaluation of fibrosis by Red Sirius or αSMA staining (Magnification x20; C). (D) CD31 and aminopeptidase P-positive blood microvessels from renal cortex samples from uni-nephrectomized and grafted kidneys three months after surgery (Magnification x40; C). Values (mean ± SEM) significantly different from those of the uni-nephrectomized kidney group are represented by **p < 0.01.

Three months after transplantation, renal IR induced vascular remodeling, as shown by an increased vascular media-to-lumen ratio (p < 0.05; Fig 5A and 5B and S1 Appendix). Tissue injuries were associated with greater urinary protein excretion by the kidney graft than uni-nephrectomized kidney group one month (125 ± 2 *vs*. 40 ± 1 mg/mmol creatinine, p < 0.01) and three months (108 ± 2 *vs*. 63 ± 2 mg/mmol creatinine, p < 0.01) following renal transplantation (Fig 2 and S1 Appendix). This correlated with the decrease in the number of vessels with a diameter < 30 μm observed at three months after transplantation (r^2^ = 0.72, p = 0.002). In addition, grafted kidneys exhibited interstitial fibrosis relative to those of the uni-nephrectomized group, as shown by red Sirius and αSMA staining (p < 0.05 for both, Fig 5C, Table 3 and S1 Appendix). Staining of endothelial cells for CD31 and aminopeptidase P supported the decrease in the number of peritubular microvessels in a graft three months after surgery (p < 0.05 for both, Fig 5D, Table 3).

**Table 3:** Summary table of semi-quantification of total cortex staining from kidneys three months after auto-transplantation.

Parameters	Uni-nephrectomized kidneys	Grafted kidneys
Alpha SMA positive tubules/field	6.23 ± 0.46	22.03 ± 0,38**
Red Sirius staining (%)	8.25 ± 0.42	21.79 ± 1.18**
Ratio of peritubular capillaries/tubules (CD31 staining)	0.81 ± 0.01	0.21 ± 0.01**
Number of capillaries/field (amino-peptidase staining)	24.34 ± 0.36	12.48 ± 0.20**

Mann Whitney test **p < 0.01

### Renal transplantation induces profibrotic and antiangiogenic pathways associated with decreased NO production

**Fig 6:**
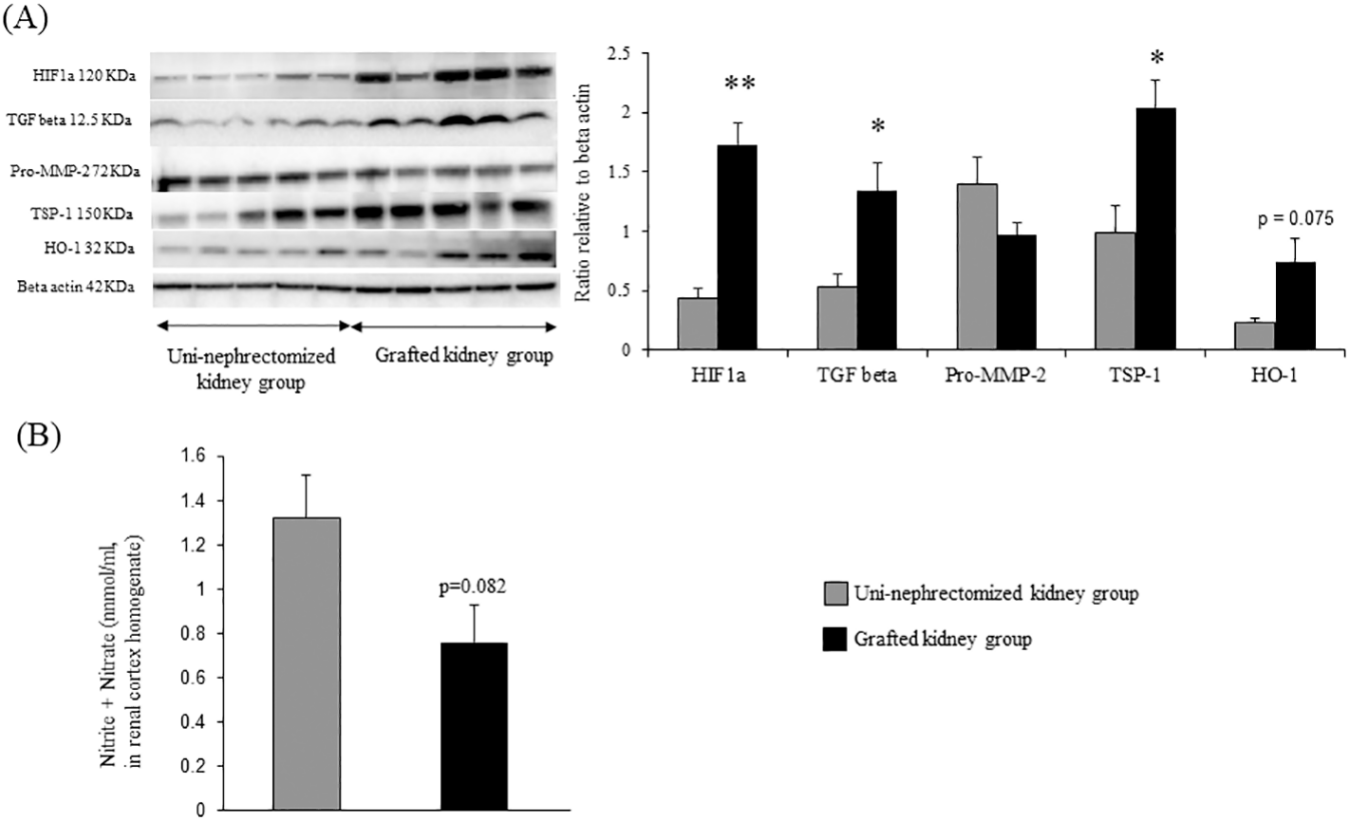
Renal transplantation induces profibrotic and antiangiogenic pathways associated with decreased NO production. Western blot analysis of proteins involved in profibrotic and antiangiogenic pathways in cortex samples from uni-nephrectomized and grafted kidneys. (B) Evaluation of NO production by quantification of nitrite + nitrate. Values (mean ± SEM) significantly different from those of the uni-nephrectomized kidney group are represented by **p < 0.01; *p < 0.05.

We investigated the expression of renal profibrotic and antiangiogenic factors to assess the pathways involved in vascular remodeling three months after transplantation. Renal transplantation was associated with a significant increase in the expression of TGFβ, the main profibrotic factor, (~2.2-fold, p < 0.05), HIF1a (~2.5-fold, p < 0.01), and TSP-1 (~2.0-fold, p < 0.05) (Fig 6A). There were also trends towards increased HO-1 expression (p = 0.075, Fig 6A) and decreased NO production (p = 0.082, Fig 6B, Table 3 and S1 Appendix) in kidney grafts relative to uni-nephrectomized kidneys.

## Discussion

Transplantation is the primary choice of treatment for end-stage renal failure. Delayed graft dysfunction and graft loss due to IR is still a major concern, despite improvements in organ preservation and immunosuppressive therapy [2, 22]. The microvascular network is the major target of IR injuries [23–25]. Previous studies have reported that microvascular graft perfusion negatively correlates with cold ischemia time and reperfusion-associated parenchymal injury [3, 26]. Here, we observed a decrease in mean vascular segment length three months after renal transplantation associated with a concomitant reduction of vascular segment volume, particularly in the outer part of the cortex, although cortical thickness was unaffected. This is the first demonstration that microcirculation is affected after transplantation due to a reduction in the number of vessels in the microvascular with a diameter < 30 μm. This finding highlights the size of targeted vessels in IR, and more generally, the involvement of IR-induced injuries in the spectrum of vascular diseases. The observed rarefaction of small vessels is supported by the sustained decrease of cortical blood flow, as assessed by laser doppler flowmetry, and the decreased density of peritubular microvessels, as assessed by CD31 staining in the cortex. Taken together, these results suggest that upstream afferent arterioles were affected by the renal transplantation procedure, thus disturbing tubular reabsorptive function. This is supported by the high urinary fractional sodium and magnesium excretion and osmolarity ratio. Such tubulopathy could be linked to an underlying reduction of blood flow in efferent arterioles and peritubular capillaries. This interpretation is consistent with a previous study in rats which showed a persistent reduction in peritubular capillary density in the cortex and outer stripe of the medulla following ischemic renal injury [5]. Efficient cortical reabsorption is highly dependent on peritubular capillaries. Thus, a reduction in their density would be expected to hamper tubular function. Indeed, the observed two to four-fold decrease in the number of small vessels was associated with a similar increase in sodium and magnesium excretion. The diameter of afferent arterioles ranges from 20 to 40 μm [27–29]. It is likely that a significant fraction of afferent arterioles was concerned by the observed rarefaction of small vessels, as our cut-off size was 30 μm, possibly explaining the reduced cortical blood flow. Finally, our results suggest an increase in sodium delivery to the *macula densa*, suggesting that the activation of tubulo-glomerular feedback can be involved in reduced cortical blood flow [27–29].

This 3D imaging approach allows for refined vessel classification and better understanding of renal graft cortical microcirculation, as it provides new parameters, such as the vascular volume fraction [9]. HR-μCT analysis has already been compared to the gold standard of light microscopy and the accuracy of this technique has been validated, strengthening the relevance of our results [15].

The impact of IR on microvascularization results from a complex interaction between increased vascular permeability, endothelial cell activation, the imbalance between vasodilatation and vasoconstriction, and the activation of the coagulation and oxidative stress response [30–32]. These factors are associated with a no-reflow phenomenon, i.e. increased impedance of microvascular blood flow after tentative reperfusion of occluded/thrombosed vessels [32, 33]. Reffelmann *et al*. reported that no-reflow can persist long after IR and predict organ failure [33], in accordance with the present results.

The results here show that the middle and inner cortex, encompassing the cortico-medullary junction, appear to be particularly sensitive to IR. This has been previously reported in other animal models, albeit such results cannot be extrapolated to human beings [5, 34]. However, the porcine model is particularly relevant, because porcine and human renal anatomy shares comparable multilobular and multipapillary architectures, whereas dogs, rabbits, rats, and mice have unilobular and unipapillary kidneys, limiting their usefulness for the study of human diseases [8, 35, 36]. Sub-regional selectivity may also be partially related to anatomical and functional differences between the outer and deep renal cortex [4]. Approximately 80% of renal blood flow is distributed to the outer half of the cortex, which contains approximately half of the glomeruli [37], and is selectively affected by decreased renal perfusion pressure [4]. The reduction of the vascular segment volume observed in the outer and middle cortex may affect the function of the renal graft. Our results show a higher number of bifurcations and a lower arborescence factor in auto-transplanted than uni-nephrectomized kidneys, suggesting that vascular remodeling is particularly marked in the outer part of the cortex three months after transplantation surgery. Higher vascular network complexity induced by IR could be associated with the renal regenerative process and angiogenesis [16, 26, 38].

Renal fibrosis has already been observed in this porcine renal autotransplantation model with a three-month follow-up, [16, 26, 38]. Fibrosis, strongly suspected of inducing chronic hypoxia, is a pivotal factor that strongly influences graft survival. We show here that it is accompanied by capillary rarefaction in the peritubular area, marked by a decrease of CD31 and aminopeptidase P-positive blood microvessels. Fibrosis also promotes vascular remodeling, as suggested by the increased media-to-lumen ratio [16, 39, 40]. These results, associated with a trend towards decrease NO production in kidney grafts, indicate that renal graft perfusion is still impaired three months after transplantation. Here, the suggestion of fibrosis by red Sirius and αSMA staining, concomitant with the activation of profibrotic pathways, such as TGF β, are likely indicative of an ongoing process. Such tissue remodeling could be involved in microvessel network modifications and higher urinary protein excretion. Indeed, we show a significant correlation between urinary protein excretion levels and microvessel rarefaction, highlighting the clinical relevance of the results. In addition, microvessel rarefaction could also be partially caused by anti-angiogenic protein activation, such as TSP-1, as also observed in this study. TSP-1 interacts with vascular endothelial growth factor, reducing vessel formation and the maturation in kidney grafts. The expression of HIF1a, a transcription factor stabilized by poor oxygen delivery, was also elevated in grafts, suggesting a hypoxic milieu that may be partially explained by renal fibrosis. This is supported by the trend towards HO-1 expression, a downstream mediator of HIF1a.

Our study was limited by the use of young pigs with a short post-transplant follow-up period relative to the human disease. In addition, anastomosis in renal veins or arteries, the keystone of the surgical transplantation procedure, could impair reconstructive flow and patency and potentially induce chronic vascular remodeling, independently of IR injury.

A preclinical model, such as ours, offers the possibility of studying various aspects of kidney preservation and developing objective protocols for organ management that are easily transposable to clinical situations. Moreover, this approach is essential for characterizing the mechanisms induced by IR on vascular networks, identifying lesions or regenerative processes, and gaining valuable insights into diagnostic and therapeutic development suited to renal graft management.

## Conclusions

This study characterized vascular remodeling following porcine kidney transplantation related to IR-induced microvascular injuries, particularly of small inner cortical vessels. Vascular remodeling consisted largely of increased vascular network complexity, seemingly linked to a regenerative process and the promotion of neovessels from existing vascular segments. This methodology could be used to investigate potential therapeutic interventions in large animal models, with the objective to minimize vascular injury in situations of IR following transplantation, and to develop future therapy towards micro-vessel preservation or angiogenesis in transplantation.

## Supporting information

S1 Appendix Supplemental data. Excel spreadsheets including results of biological parameters and data obtained from processing of high resolution micro-computed tomography acquisition images of renal cortex biopsies.
(XLSX)
